# Potential Applications of *Zingiber officinale*, *Clitoria ternatea*, and *Coccinia grandis* for Enhancing the Functionality of Set Yogurts

**DOI:** 10.1002/fsn3.70475

**Published:** 2025-06-27

**Authors:** Yohan Jayaweera, Mayumi Silva, Jayani Chandrapala, Rumesh Liyanage, K. G. L. R. Jayathunge, Amali U. Alahakoon

**Affiliations:** ^1^ Department of Biosystems Technology, Faculty of Technology University of Sri Jayewardenepura Homagama Sri Lanka; ^2^ School of Science, STEM College RMIT University Melbourne Victoria Australia

**Keywords:** antioxidant activity, fortified yogurts, FTIR, functionality, microstructure

## Abstract

The growing consumer interest in health‐promoting foods has encouraged the development of yogurt fortified with plant‐based additives. This study focused on the effect of aqueous extracts from ginger ( *Zingiber officinale* ) rhizome, blue butterfly pea ( *Clitoria ternatea* ) flower, and ivy ( *Coccinia grandis* ) leaf on the physicochemical and functional characteristics of yogurt, with a particular emphasis on understanding their interaction with milk proteins. Incorporating all the plant extracts enhanced consumer satisfaction regarding color, odor, texture, taste, mouthfeel, and aftertaste. The ivy leaf and blue butterfly pea flower fortification significantly reduced the pH values and increased the acidity compared with the unfortified yogurt on Day 1. This effect is further validated by lactic acid bacteria count results, suggesting that these extracts can promote the growth of lactic acid bacteria. The elevated total phenolic and antioxidant activities in the fortified yogurts validated the fortification process. FTIR analysis confirmed a possible change in the milk protein secondary structure due to the plant's phytochemicals. SEM analysis confirmed that these structural changes, depending on the plant extract, could be either beneficial or detrimental. Specifically, ginger and blue butterfly pea flower extract fortification reduced protein aggregate size and formed more compact and denser gel networks, resulting in lower syneresis and a higher viscosity yogurt structure compared to the unfortified yogurt. In contrast, ivy leaf extract fortification formed a less dense and less compact structure with large aggregates, resulting in higher syneresis and lower viscosity. The overall findings suggest promising opportunities for the yogurt industry with ginger and blue butterfly pea flowers while highlighting the need for in‐depth research on ivy leaf extract‐fortified yogurts.

## Graphical abstract

In this study, aqueous extracts of ginger, blue butterfly pea flower, and ivy leaf were fortified to the yogurts and evaluated the sensorial, physicochemical, and functional characteristics of yogurt along with the protein interactions. Results revealed that the fortification of all the plant extracts had a significant impact on the sensorial, physicochemical, antioxidant, and structural properties of the yogurts.

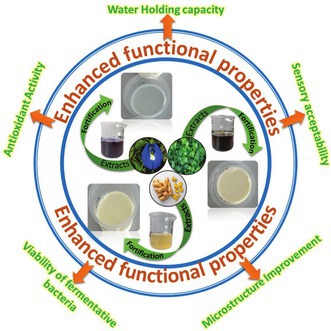

## 1 Introduction

Functional foods are generally designed to provide additional health benefits beyond the basic nutritional functions of foods. These benefits include the prevention of nutrition‐related and non‐nutrition‐related diseases, improvements in physical and mental well‐being, and vitality and longevity (Granato et al. 2020). The design and development of functional food products have been associated with incorporating one or more functional ingredients, bioactives or nutraceuticals, into the food‐based delivery medium (Day et al. 2009). In this context, yogurt, which is a very popular fermented dairy product, stands out as a perfect medium for such a fortification.

Yogurt is produced with the action of two main lactic acid bacteria ( *Lactobacillus delbrueckii* subsp. *bulgaricus* and *Streptococcus thermophilus* ) (Aamir et al. 2023). It enriches protein, minerals, vitamins, and omega‐3 fatty acids, and these nutritional values have led to a global increase in the consumption of yogurts (Le Roy et al. 2022). Moreover, due to the growing interest of modern consumers in healthier food options and their preference for fewer adverse effects, the development of yogurt with plant additives has become a trend. Consequently, various plants and their parts, such as cinnamon (Jiménez‐Redondo et al. 2022), fennel extract (Atwaa et al. 2022), strawberry pulp (Jaster et al. 2018), and Moringa extracts (Zhang et al. 2019) have been incorporated with yogurt to develop fortified yogurts. In general, fortification with these plants modifies the sensory profiles (flavor, taste, color, and aroma) and enhances the nutritional content, digestive health, and antioxidant capacities of yogurts (Wazzan 2024).

There is significant potential in using underutilized plants or herbs that possess unique nutritional profiles and bioactive compounds for the fortification process of yogurt. Among these plant sources, ginger ( *Zingiber officinale* ), ivy leaves ( *Coccinia grandis* ), and blue butterfly pea flowers ( *Clitoria ternatea* ) exhibited significant health‐promoting properties such as antioxidant, anti‐cancer, antidiabetic, anti‐inflammatory, antimicrobial, antifungal, hepatoprotective, and neuroprotective (Pekamwar et al. 2013; Wang et al. 2014). Furthermore, these plants are rich in phytochemicals with significant medicinal benefits and offer positive potential for sustainable, organic, and clean‐label products due to their easy extraction process, minimal processing requirements, being free of synthetic additives, and easy cultivation practices. Moreover, the long history of usage in traditional medicine, combined with the recent trend in modern medicinal and functional food applications, demonstrates their suitability and dependability as natural fortifying ingredients; however, thorough investigation on their effect with food components is not addressed well. These characteristics present them as appealing options in research and industry, as consumer attitudes shift toward multi‐benefit, natural additives (Sbardelotto et al. 2023).

With the advantages of health‐promoting activities of these plants and recent trends, studies have been conducted on fortifying ginger and blue butterfly pea flowers in yogurt to some extent. For instance, ginger fortification has been shown to significantly enhance the antioxidant properties and sensory attributes in terms of flavor, texture, and color of the yogurts (Larasati et al. 2018). Aamir et al. (2023) have found that the fortification of 0.5%, 1%, 1.5%, and 2% of ginger powder significantly improved the acidity, water‐holding capacity, total phenolic content, sensory acceptability, and nutritional composition (protein) while minimizing the yeast and mold count. In addition, Gamage et al. (2024) reported that the fortification of 1% and 2% (w/v) blue butterfly pea flower extracts exhibited stable pH, acidity, syneresis conditions, higher anthocyanin stability, higher DPPH radical scavenging activity, and FRAP activity but lowered lactic acid bacteria viability compared with the unfortified yogurts. Furthermore, the other studies found that blue butterfly pea flower fortified yogurt has improved antioxidant (DPPH and total phenolic content), textural (syneresis and viscosity), and sensory characteristics in terms of color, flavor, mouthfeel, and overall acceptability (Kumar et al. 2023; Sutakwa et al. 2021).

However, these results can greatly depend on factors such as fortifying ingredient type, concentration levels, geographical location, and weather (Pereira et al. 2024). The conformational changes and microstructure of these fortified yogurts still need to be explored for a thorough understanding. In addition, ivy leaves have been consumed as a vegetable in Sri Lanka for centuries, yet their potential for incorporation into fortified yogurts remains unexplored (Putra et al. 2021). Therefore, this study assessed the fortification of yogurt with ginger, ivy leaf, and blue butterfly pea flowers, thoroughly evaluating their physicochemical, antioxidant, structural, conformational changes, and microstructural properties to comprehensively understand their interactions within the fortified yogurt matrices.

## 2 Materials and Methods

### 2.1 Materials

Fresh cow milk was purchased from the local market in Sri Lanka. Commercial lactic acid bacteria ( *S. thermophilus* and *L. delbrueckii* subsp. *bulgaricus*) culture was obtained from Chr. Hansen Laboratories (Copenhagen, Denmark). Matured ginger ( *Zingiber officinale* ) rhizome, blue butterfly pea ( *Clitoria ternatea* ) flower, and ivy ( *Coccinia grandis* ) leaves were obtained from selected cultivation sites in the western province of Sri Lanka and authenticated from the National Herbarium at Peradeniya, Sri Lanka. Then, the plant samples and rhizomes were cleaned with distilled water and oven‐dried (NL 1017 X/007, Selangor, China) at 45°C until a constant weight was obtained. Thereafter, the plant materials were ground using a domestic grinder (Philips HL7756/00, India) until they became a fine powder (mesh size 30). Then, the obtained powder samples were vacuum packed and stored at −24°C until further usage.

### 2.2 Preparation of Plant Extracts

The aqueous extracts of all plant materials were obtained according to the method described by Farhan et al. (2020) with slight modification. Plant powder (5 g) was mixed with 100 mL of distilled water, and then the solution was heated and stirred using a hot magnetic stirrer (F800, China) at 100°C for 15 min. Thereafter, each extract was filtered using filter paper (Whatman No 1) and centrifuged at 10,000× g (Sorvall ST 8R, Germany) for 10 min at 4°C. The collected supernatants were stored at 4°C in the dark until further analysis.

### 2.3 Preparation of Plant Extract‐Fortified Yogurts

Initially, 5% (w/v) skim milk powder was added to the milk to increase the total solid content to obtain a better body and texture for yogurt. Then, the plant extracts were incorporated into milk at different fortification levels (1%, 2%, and 3% v/v), which were selected by a preliminary investigation such as evaluating antioxidant capacity that needed to exhibit a significant difference, sensory evaluation, and shelf‐life assessment. Thereafter, the milk was pasteurized at 72°C for 15 s, cooled to 42°C, and incubated with the starter culture (2% w/v) until the pH of the sample became 4.6 (about 4 h). Then, the samples were stored at 4°C for 21 days and evaluated for physicochemical, microbiological, total phenolic, and antioxidant properties at 7‐day intervals after selecting the suitable fortification level for each treatment by sensory evaluations.

The yogurt fortified with aqueous extracts of ginger rhizome, blue butterfly pea flower, and ivy leaves was denoted as GY, BY, and IY, respectively, whereas the unfortified yogurt that is used as the control group (0%) was denoted as CY.

### 2.4 Sensory Evaluation of Plant Extract‐Fortified Yogurts

All fortified yogurts underwent a sensory evaluation on the 1st day of storage. A group of 30 semi‐trained sensory panels (15 males and 15 females) from the Faculty of Technology, University of Sri Jayewardenepura, Sri Lanka, was selected. Color, aroma, texture, flavor, mouthfeel, aftertaste, and overall acceptability of fortified yogurts were evaluated using the 9‐hedonic scale (1 point‐extremely dislike to 9 points‐extremely like) (Tami et al. 2022). After selecting the most preferred fortification level for each yogurt, the rest of the analysis was conducted on those selected concentrations of fortified yogurts. Before the sensory analysis, the sensory panel was trained by dilution test, difference threshold test, taint potential test, triangle test, and duo‐trio test. One‐way ANOVA followed by post hoc Tukey's multiple comparison test was used to determine the significant difference (*p* < 0.05).

### 2.5 Physicochemical Properties of the Yogurts

#### 2.5.1 Proximate Analysis of the Yogurts

Moisture content (gravimetric method), crude protein content (Kjeldhal method), fat content (Gerber method), ash content (gravimetric method), and carbohydrate content (phenol sulfuric method) were determined by the methods of AOAC (2005).

#### 2.5.2 pH and Titratable Acidity

The pH value of fortified yogurts was determined using a pH meter (HI83141, Hanna Instrument) after calibration with standard pH buffers (4.0 pH and 7.0 pH). For titratable acidity, yogurt samples were diluted with distilled water at a 1:1 ratio (v/v) and titrated with NaOH (0.1 N) until a pale pink color was obtained with a phenolphthalein indicator (0.1% w/v). Then, the acidity of the yogurts was calculated according to Equation (1), and the results were expressed as the percentage of lactic acid (%) (Nazari et al. 2023).

(1) $$\text{Titratable acidity}\;\left(\%\right)=\left(\text{NaOH volume}\;\left(\mathrm{mL}\right)\times 10\times 0.009\times 0.1/W\right)\times 100$$

where *W* = weight of the yogurt sample.

#### 2.5.3 Syneresis of the Yogurts

Syneresis of the yogurts was evaluated according to the method described by Bulut et al. (2021). An amount of 10 g of yogurt samples was added to a Falcon tube and centrifuged (Sorvall ST 8R, Germany) at 7870× g and 4°C for 10 min. Then, the obtained clear supernatant was poured and weighed. The percentage of syneresis was calculated using the following equation: (2).
(2)

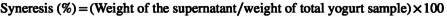




#### 2.5.4 Viscosity of the Yogurts

The viscosity of all the yogurts was determined with a viscometer (BDV—9S, China), using spindle No. 4 at 4°C for 5 min at 30 rpm. The results were expressed in mPa.s (Pavalakumar et al. 2021).

#### 2.5.5 Color of the Yogurts

The color of the yogurt was evaluated using a digital colorimeter (Lovibond LC 100, United Kindom). After the calibration of the instrument, the values of lightness (L*), redness (a*), and yellowness (b*) were obtained from 9 different points of each yogurt sample.

### 2.6 Fourier Transform Infrared Spectroscopy (FTIR) Analysis

The FTIR analysis was conducted according to the method described by (Gunathunga et al. 2024). Initially, freeze‐dried yogurt samples were homogenized thoroughly, and then a small amount was applied to the attenuated total reflectance (ATR). Then, freeze‐dried yogurt samples were analyzed using an FTIR spectrophotometer (Bruker Vertex 80, USA). Infrared spectra of all the yogurt samples were obtained in the 4000–400 cm^−1^ region, with a resolution of 4 cm^−1^ after 16 scans, after background subtraction. Two FTIR spectral acquisitions were carried out for each yogurt sample. The obtained results were normalized, smoothed, obtained second derivative, and analyzed using OriginPro software (version SR1 b9.5. 1. 195, USA). The second derivative spectra were examined in the 1600–1700 cm^−1^ region, corresponding to the Amide I band, which provides insights into protein secondary structures. Band assignments were made based on previous studies, identifying β‐sheets (1610–1642 cm^−1^ and 1688–1697 cm^−1^), random coils (1644–1648 cm^−1^), α‐helix structures (1651–1653 cm^−1^), large loops (1656–1658 cm^−1^), and β‐turns (1667–1684 cm^−1^) (Grewal et al. 2018; Usoltsev et al. 2019).

### 2.7 Scanning Electron Microscopy (SEM) Analysis

The freeze‐dried yogurt samples were placed in a desiccator to remove any moisture completely until they obtained constant weight. Thereafter, all the samples were fixed on the stub and coated with a thin layer of gold using sputtering equipment before the analysis (Ismail et al. 2020). Then, the microstructure of all the coated yogurt samples was analyzed by taking five images for each sample using a scanning electron microscope (ZEISS EVO 18, Germany) at 15,000× magnification with an excitation voltage of 10 kV and 10.5 mm working distance.

### 2.8 Microbial Analysis of the Yogurts

*Streptococcus thermophilus* , *L. delbrueckii* subsp. *bulgaricus*, yeast, and mold counts were evaluated according to the method described by Ibrahim et al. (2019). Briefly, 1 mL of each yogurt dilution was mixed with 9 mL of peptone water and serial dilutions. Then, 1 mL of the samples was poured into the M17 agar ( *S. thermophilus* ), MRS agar ( *L. delbrueckii* subsp. *bulgaricus*), and PDA (yeast and mold). Then, the M17 plates were incubated at 42°C for 48 h, and MRS and PDA plates were incubated at 37°C for 72 h. The results were expressed as a log number of colony‐forming units per mL (Log CFU mL^−1^).

### 2.9 Total Phenolic Content and Antioxidant Activity of Fortified Yogurts

#### 2.9.1 Preparation of Yogurt Aqueous Extracts

The aqueous extracts of yogurts were obtained according to the method described by Nazari et al. (2023) with very slight modifications. Yogurt (20 g) was mixed with 5 mL of distilled water, and then the pH of the mixture was adjusted to pH 4.0 using 0.1 M HCl solution. Thereafter, the mixture was placed in a water bath for 10 min at 45°C. Then, the mixture was centrifuged (Sorvall ST 8R) at 10,000× g at 4°C for 10 min. The supernatant was separated, and the pH of the supernatant was adjusted to pH 7.0 with the solution of 0.1 M NaOH and again centrifuged at 10,000× g at 4°C for 10 min. Then, the supernatant was separated and stored at −24°C in the freezer. This supernatant was used to evaluate the antioxidant activity DPPH (2,2‐diphenyl‐1‐picrylhydrazyl) and ABTS (2,2‐azino‐di‐3‐ethylbenzothialozine‐sulphonic acid) and total phenolic content of the yogurts.

#### 2.9.2 Total Phenolic Content of Fortified Yogurts

Total phenol content was analyzed spectrophotometrically using the Folin–Ciocalteu method (Ghafoor et al. 2020). A 10% Folin–Ciocalteu reagent (5 mL) and 7% Na_2_CO_3_ (4 mL) were mixed with 1 mL of yogurt extract in a test tube. The obtained blue‐colored mixture was shaken well and incubated in a water bath for 30 min at 40°C. Then, the absorbance was measured at 760 nm using a spectrophotometer (G10S, USA) against the blank, and total phenolic content was calculated based on the standard curve of gallic acid (20 to 100 μg mL^−1^), and the results were expressed as μg gallic acid equivalent per mL (μg GAE mL^−1^).

#### 2.9.3 DPPH Radical Scavenging Activity of Yogurts

The DPPH free radical scavenging activity of the yogurt extracts was measured by the DPPH assay procedure described by Hatano et al. (1988) with slight modifications. One thousand microliters of methanolic DPPH solution was added to 2 mL of yogurt extract and shaken well. Then, the mixture was incubated at 25°C for 30 min, and the absorbance of the solutions was measured at 517 nm in a spectrophotometer (G10S, USA). The obtained absorbance was calculated using the standard curve of ascorbic acid, and the results were expressed as μg ascorbic acid equivalent per mL (μg AAE mL^−1^).

#### 2.9.4 ABTS Radical Scavenging Activity of Yogurts

ABTS activity for yogurt extracts was determined according to the method by Mehmood et al. (2019). Briefly, ABTS radical stock solution was prepared by adding ABTS (7 mM) with K_2_SO_8_ and storing the mixture in the dark for 16 h. The ABTS radical solution was diluted with methanol to obtain an absorbance of 0.70 ± 0.02 at 734 nm. An 80 μL yogurt extract was mixed with 3.92 mL of ABTS radical working solution in the dark, and absorbance was measured at 734 nm using a spectrophotometer (G10S, USA) after keeping it for 6 min at 25°C, and the results were expressed as μg ascorbic acid equivalent per mL (μg AAE mL^−1^). The standard curve was prepared using ascorbic acid (15 to 120 μg mL^−1^).

### 2.10 Statistical Analysis

All the analysis was evaluated at least in triplicates, and the results are shown as mean ± standard deviation. The significant difference between the means was evaluated by one‐way ANOVA and post hoc Tukey's test with a 95% confidence level (*p* < 0.05) using the SPSS software version 21.

## 3 Results and Discussion

### 3.1 Sensory Evaluation of Plant Extract‐Fortified Yogurt

**FIGURE 1:**
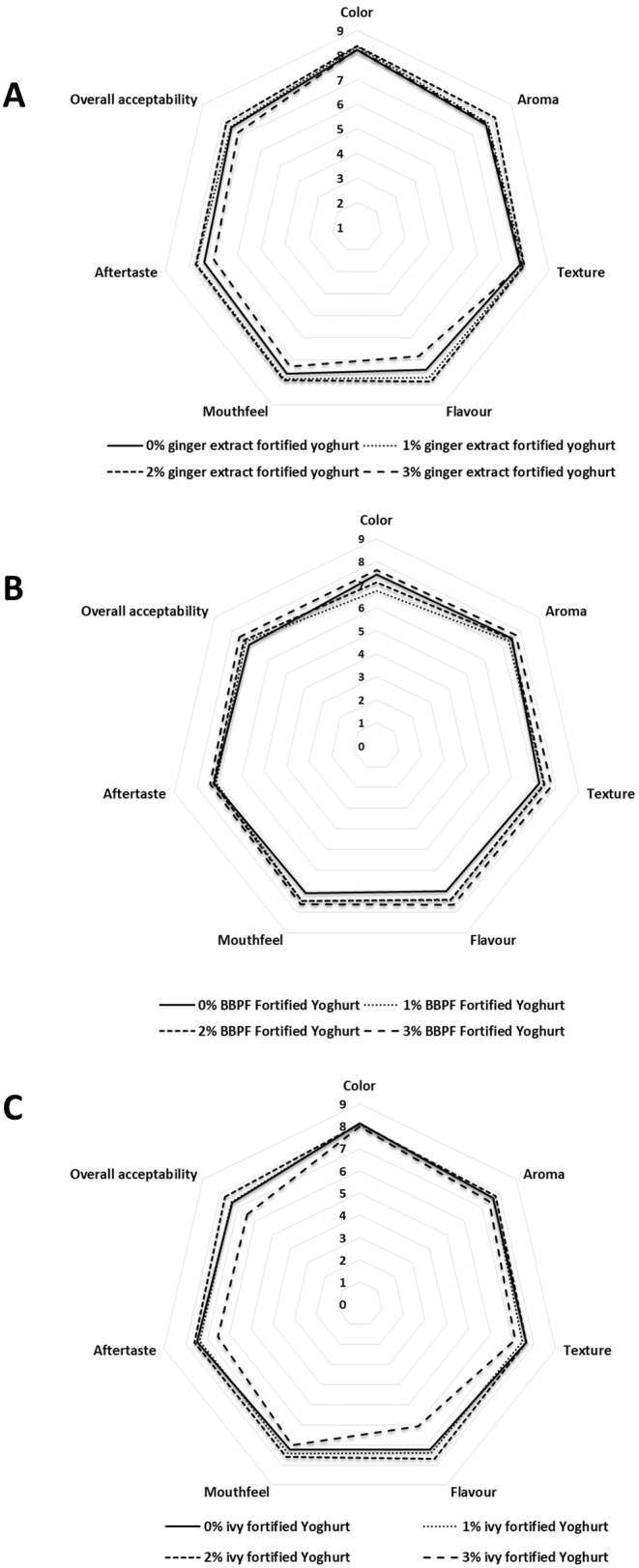
Web chart for the sensory profile of ginger (A), blue butterfly pea flower (B), and ivy leave (C) aqueous extract‐fortified yogurt at 0%, 1%, 2%, and 3% concentrations on day 1 at 4°C (one‐way analysis of variance followed by Tukey post hoc test).

The sensory analysis results of fortified yogurts with ginger extract, blue butterfly, and ivy leaf extract are shown in Figure 1A–C, respectively. None of the treatments showed a significant difference in evaluated sensory parameters compared with the CY. However, 2% GY, 2% IY, and 3% BY exhibited numerically the highest scores for all the evaluated sensory attributes compared to the CY. According to the results, it is clear that incorporating these plant extracts has the potential to improve the sensory properties of the fortified yogurts, and this could be due to the different phytochemicals available in ginger (gingerol, shogaols, paradols, and zingerone, like major compounds) (Spence 2023), blue butterfly pea flower (ternatins, quercetin and kaempferol derivatives) (Kusuma et al. 2024), and ivy leaves (cathinone, heptacosane, gallic acid, and tritetracontane) (Hossain et al. 2024). Even though the plant extracts improved the sensory properties, the observed nonsignificant results during the sensory analysis could be attributed to individual variations in sensory perception among panelists. The variability in how participants perceived attributes such as flavor, texture, and color might have reduced the statistical significance. Additionally, the concentration levels of fortifying extracts may not have been strong enough to produce noticeable differences in sensory attributes compared with the CY. Similarly, Aydın et al. (2025) reported that spraying of aqueous extract of olive leaf at 0.4% and 0.6% fortification levels on the surface of yogurt after fermentation showed the highest consumer acceptability with no significant difference compared with the unfortified yogurts, while exhibiting excellent antimicrobial activity against yeast and mold without interfering with the lactic acid fermentation.

### 3.2 Proximate Composition of Yogurts

The proximate composition (moisture, protein, fat, carbohydrate, total solids, and ash content) of all the yogurts is shown in Table 1. The results showed no differences in the proximate composition of GY, BY, and IY compared to CY, except for ash content. The improved ash content could be due to the mineral component of the plant extracts (Edogun et al. 2024). The minimal impact of ginger, blue butterfly, and ivy leaf extracts on the nutritional profile of yogurt offers a promising opportunity to improve its functional properties without compromising its basic nutritional value. Similarly, Pavalakumar et al. (2021) reported that the addition of cinnamon (100 ppm) and turmeric oleoresins (100 ppm) had no significant impact on the proximate composition of fortified yogurts. However, in contradiction to our results, Aamir et al. (2023) reported that adding ginger powder at concentrations ranging from 0.5% to 2% to yogurt resulted in an increment in the total solids content and protein content. The differences in results can primarily be attributed to the variation in the type and form of ginger used, which interact differently with yogurt ingredients. Additionally, concentration levels play a significant role, as ginger powder contains higher concentrations of nutrients than extracts. Moreover, variations in the yogurt formulation itself can influence how the fortifying ingredients affect the final proximate composition, as different base compositions may respond differently to fortification.

**TABLE 1:** Proximate composition of the unfortified and fortified set yogurts.

	CY	GY	BY	IY
Moisture	75.65 ± 0.08	75.75 ± 0.08	75.78 ± 0.13	75.80 ± 0.33
Protein	3.43 ± 0.02	3.44 ± 0.02	3.45 ± 0.02	3.44 ± 0.02
Fat	3.13 ± 0.05	3.16 ± 0.05	3.13 ± 0.09	3.12 ± 0.04
Carbohydrate	16.53 ± 0.02	16.54 ± 0.01	16.54 ± 0.01	16.53 ± 0.01
Ash	0.96 ± 0.01^b^	0.98 ± 0.01^a^	0.98 ± 0.01^a^	0.98 ± 0.01^a^
Total solid	24.35 ± 0.08	24.25 ± 0.08	24.03 ± 0.06	24.20 ± 0.33

*Note:* Each data point represents the mean ± SD (*n* = 9); The means without the letters are not significantly different from each other, ^a,b^Different superscripts within the same row differ significantly (*p* < 0.05) (one‐way analysis of variance followed by Tukey post hoc test).

Abbreviations: BY, blue butterfly pea flower fortified yogurt; CY, unfortified yogurt; GY, ginger fortified yogurt; IY, ivy leaf fortified yogurt.

### 3.3 Physicochemical Properties of Yogurts

#### 3.3.1 pH and Titratable Acidity

**FIGURE 2:**
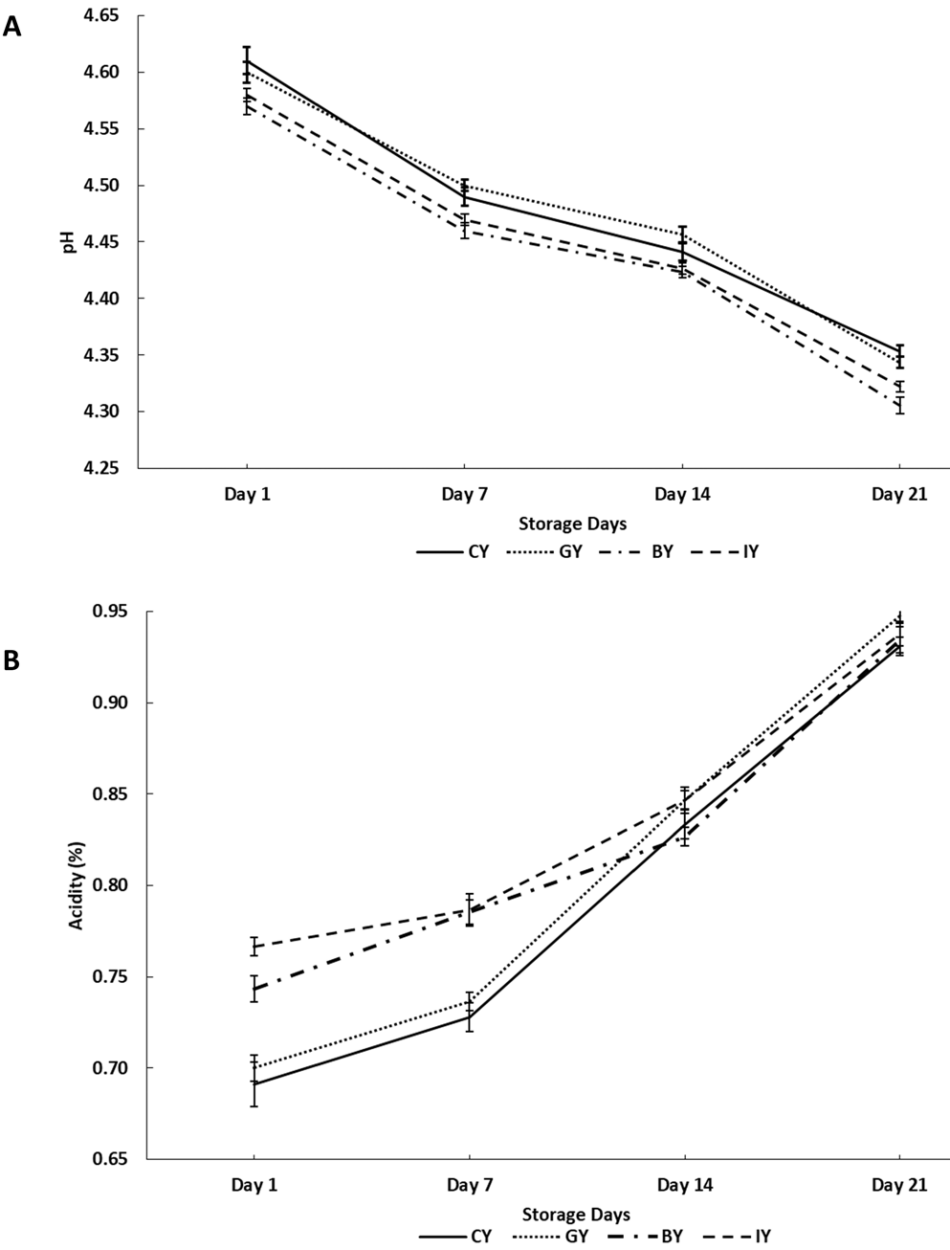
pH (A) and acidity (B) values in unfortified yogurt (CY), ginger fortified yogurt (GY), blue butterfly pea flower fortified yogurt (BY), and ivy leaf fortified yogurt (IY) during the 21‐day storage (one‐way analysis of variance followed by Tukey post hoc test).

The change of pH and acidity of fortified and unfortified yogurts during storage is shown in Figure 2A,B, respectively. According to the results on Day 1, GY exhibited a nonsignificant pH reduction and acidity increment compared to CY. However, both BY and IY exhibited significantly lowered pH and increased acidity compared to CY on Day 1. This pH reduction and acidity increment could be attributed to the boosted metabolic activity of lactic acid bacteria due to the essential nutrients in butterfly pea flower and ivy leaves, such as carbohydrates, proteins, vitamins, minerals, and phenolic compounds, including gallic acid, vanillic acid, protocatechuic acid, and anthocyanins (Pacheco‐Ordaz et al. 2018; Vivas et al. 1997). Moreover, this argument is further supported by the results of the viable count of this study (Table 3), which showed enhanced lactic acid bacteria count in BY and IY on Day 1. These nutrients allow the microbes to continuously grow and produce more lactic acid by fulfilling their carbon and nitrogen source requirements. This lactic acid content eventually results in a lowered pH level and a more acidic yogurt medium (Abdullah et al. 2023). These enhanced acidity and lower pH conditions within the recommended levels would be a great opportunity for the yogurt industry to improve microbial safety and reduce the cost of synthetic preservatives. At the same time, these conditions could be more beneficial in optimizing fermentation kinetics and resulting in better protein coagulation with better textural properties (Basha et al. 2025). Similar pH reduction and acidity increments were observed in cinnamon and cumin seed extract‐fortified yogurts (Shori 2020) and flaxseed powder‐fortified yogurts (Marand et al. 2020).

During the storage, the pH values of all the fortified and CY were significantly reduced, whereas the acidity of yogurt significantly increased throughout the storage period. These pH reductions and acidity increments over the storage are attributed to the production of lactic acid by the fermentative lactic acid bacteria in the yogurts (Hoxha et al. 2023). Moreover, the BY and IY showed significantly lower pH values compared with CY throughout the storage.

#### 3.3.2 Syneresis of the Fortified Yogurts

**FIGURE 3:**
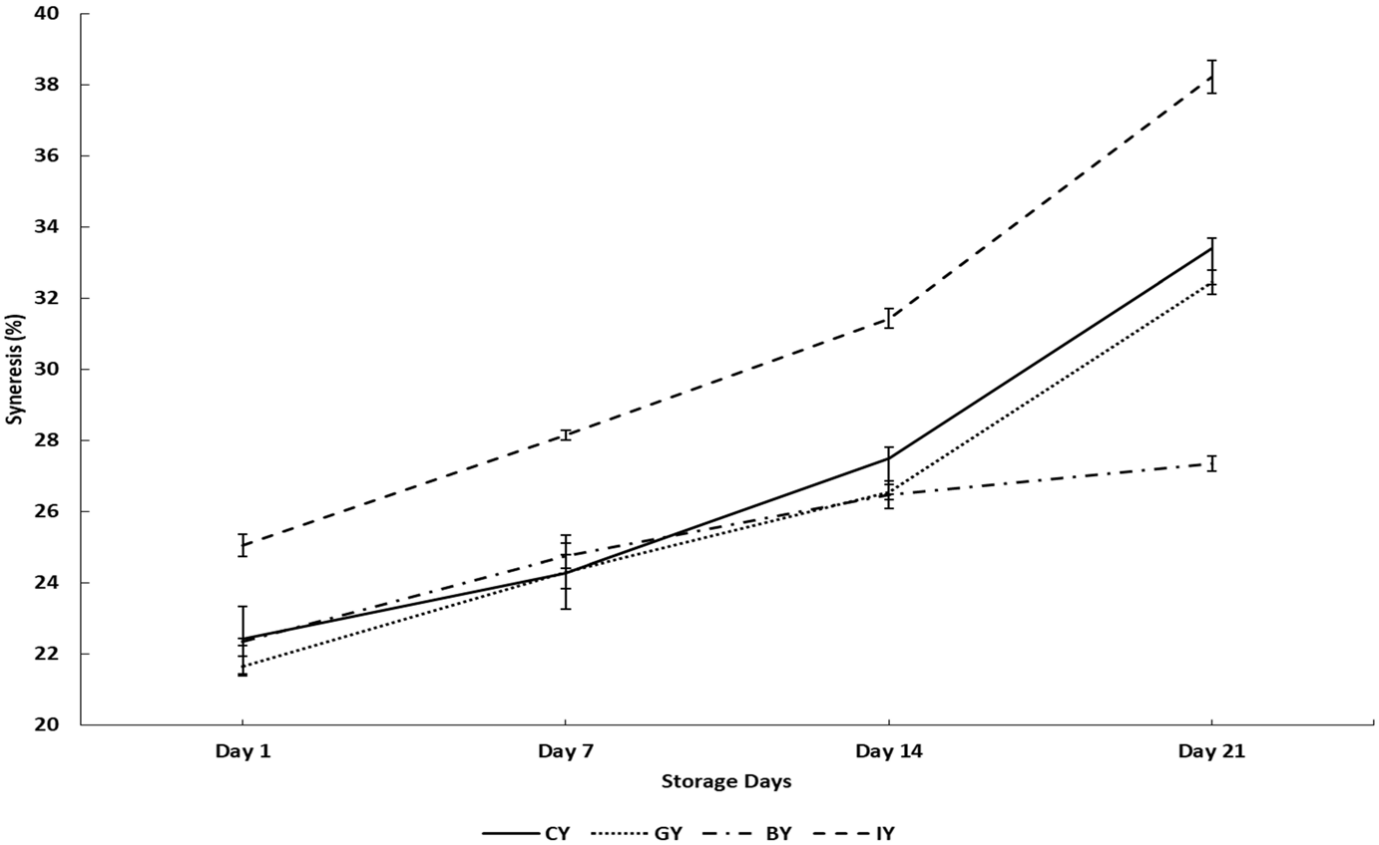
Syneresis values in the unfortified yogurt (CY), ginger fortified yogurt (GY), blue butterfly pea flower fortified yogurt (BY), and ivy leaf fortified yogurt (IY) during the 21‐day storage (one‐way analysis of variance followed by Tukey post hoc test).

The syneresis behavior during cold storage in fortified yogurts is depicted in Figure 3. The GY exhibited significantly the lowest syneresis value compared with the CY on Day 1, and this could be associated with the phenolic compound interaction with the milk protein creating a stable protein network, which can hold water, reducing the syneresis (El‐Gammal et al. 2017). These results were further supported by the microstructure analysis of this study (Figure 7), which showed a more compact and denser protein structure in GY, which eventually contributes to lower syneresis in yogurts and higher water‐holding capacity (Qiu et al. 2021). Similarly, a syneresis reduction was reported in fortified yogurts with mint extract, dragon fruit peel extract, and moringa extract due to the phenolic and protein interaction (Zahra and Mehmood 2023; Lisak Jakopović et al. 2022). However, BY did not exhibit a significant syneresis change compared with the CY on Day 1. According to Vuong and Hongsprabhas (2021), higher acidity and lower pH conditions are essential to alter the milk protein and anthocyanins interaction (Vuong and Hongsprabhas 2021). However, the low acidity and higher pH conditions in BY on Day 1 may have delayed the interaction between anthocyanins and milk proteins.

Further, the syneresis of the IY was significantly increased on Day 1 compared with the CY, but no visible layer separation was observed in the IY during the storage. According to the studies, the phenolic‐protein interaction could vary based on the type of phenolic compounds, the structure of phenolic compounds, and the bond types (Han et al. 2019). In detail, the phenolic compounds have a greater affinity to bind with casein protein rather than whey, and this could be a major reason for the changes in the structural properties of yogurt upon incorporation of plant extracts (Han et al. 2019). For instance, green tea, cranberry, and grape extracts also showed a higher potential to bind with milk protein (casein) (Han et al. 2019). Furthermore, these protein–polyphenol interactions are formed through covalent and noncovalent bonds (hydrogen bonds, hydrophobic interactions, and ionic bonds) (Ma et al. 2024). As a result, the casein aggregate size could change depending on the incorporated extract type (Han et al. 2019). This could explain the observed differences in microstructural changes and syneresis in GY, BY, and IY compared to CY. Since different plants contain different phytochemical profiles, the ivy leaves might contain some polyphenols, which can interact with the milk proteins and could affect the gel structure negatively. As a result, it could weaken the bonds between protein and water molecules, resulting in syneresis increment (Zahra and Mehmood 2023).

However, further studies should be conducted to get a better understanding of the mechanism of interaction between milk protein and phenolic compounds in ivy leaf. A similar syneresis increment was observed in coriander extract‐fortified yogurts (Zahra and Mehmood 2023). However, the lowered syneresis condition in the GY and BY could be more beneficial for the dairy industry in terms of efficient raw material management by reducing the separation of whey, less waste is produced, which allows for greater resource management and possibly whey valorization (Soumati et al. 2023). Furthermore, this syneresis reduction reduces the gelatin‐like agents' requirements, which is also beneficial for reducing the production cost that leads to higher profit margins while strengthening consumer acceptability by maintaining texture and appearance, like important sensory properties (Bong and Moraru 2014).

Syneresis values of all the fortified yogurts and CY significantly increased over the storage time, and the highest syneresis of each yogurt was observed on Day 21. This syneresis increment during the storage could be associated with the pH reduction, which encourages the contraction of the casein micellar matrix, increasing whey separation (de Moura et al. 2019). A similar type of observation was reported by Shokery et al. (2017), who found an increase in syneresis during storage in fortified yogurts with 1% green tea and 0.9% moringa leaves extracts. However, the BY exhibited the lowest syneresis value compared with the CY on Day 21. This could be due to the interaction between milk protein and rich anthocyanin in blue butterfly pea flower extract (Vuong and Hongsprabhas 2021). Accordingly, it was reported that increasing acidity and lowering pH conditions significantly increase the interaction between milk protein and anthocyanins (Vuong and Hongsprabhas 2021). According to the finding, low pH levels significantly unfold the protein secondary structure and increase its binding affinity with anthocyanin (Ren et al. 2021). This could be the reason for observing a significantly lower syneresis rate in BY at the end of the storage.

#### 3.3.3 Viscosity of the Fortified Yogurts

**FIGURE 4:**
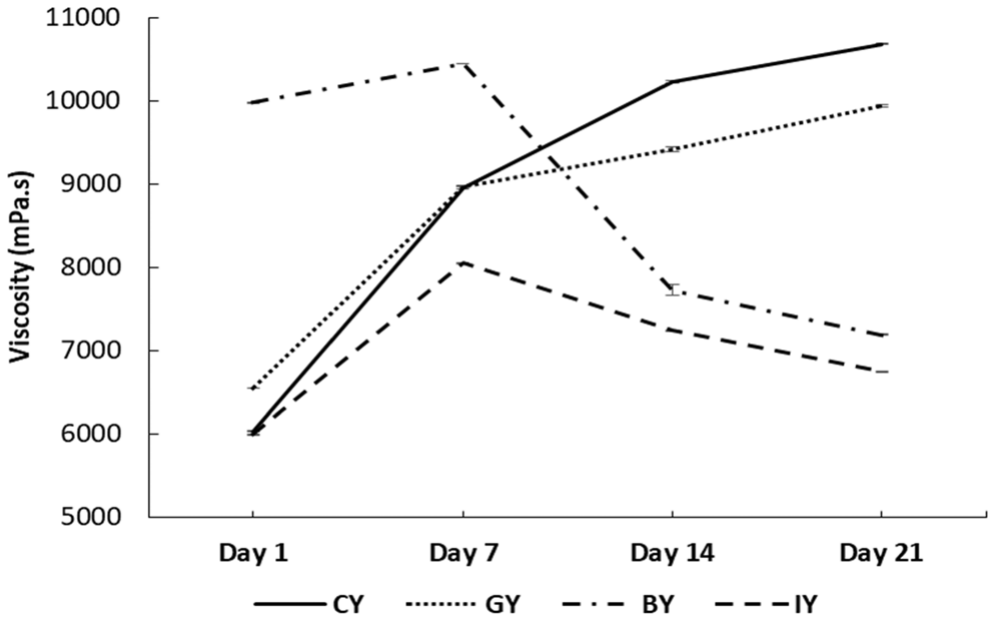
Viscosity values in CY (unfortified yogurt), GY (ginger fortified yogurt), BY (blue butterfly pea flower fortified yogurt), and IY (ivy leaf fortified yogurt) during the 21‐day storage (one‐way analysis of variance followed by Tukey post hoc test).

Figure 4 illustrates the viscosity of the fortified yogurts during storage. The BY obtained the highest viscosity among all the yogurt treatments on Day 1, which was significant. Further, GY also exhibited a significantly higher viscosity value than CY. This viscosity increment in BY and GY could be due to the interaction between yogurt proteins and phenolic compounds of ginger and blue butterfly pea flower extracts. These phenolic compounds and protein interactions develop a more stable protein gel structure, eventually exhibiting a thicker and more viscous yogurt medium (Salehi et al. 2021). This could be a reason for observing higher consumer acceptability for the texture and mouthfeel of GY and BY. Similarly, previous studies showed that the addition of pennyroyal and nano‐encapsulated pennyroyal extracts (750 and 1000 ppm) and olive leaf extracts (0.1% to 0.4%) to yogurt resulted in improved viscosity (Moghadam et al. 2021; Cho et al. 2020).

In contrast, IY exhibited a significantly low viscosity value compared to the CY on Day 1, and this could be associated with the observed syneresis increment in IY. In other words, the developed phenolic and milk protein interaction in IY affected the gel structure negatively, which caused higher syneresis and low water‐holding capacity, which may lessen casein molecules' ability to bind water in the yogurt gel matrix (Oliveira et al. 2015). This phenomenon caused a decrease in the overall thickness and consistency of the yogurts (Agyemang et al. 2020).

The viscosity increment over storage in both GY and CY could be the result of continuous protein–protein interaction in acid casein gel, wherein the bonds between the proteins rearrange as a result of the decreasing pH of yogurts during cold storage, finally contributing to increasing the gel firmness both during and after fermentation (Ozer et al. 1998). Similarly, the viscosity increment was observed with the storage time in rhubarb, grape seed, thyme, green tea, and mint extracts fortified yogurts (Bulut et al. 2021). However, the viscosity of BY and IY exhibited a decreasing trend after Day 7 and exhibited 32.65% and 36.83% viscosity reduction, respectively, on Day 21 compared to CY. This could be due to the starting of the protein gel structure breakdown in BY and IY, with the bioactive compound degradation in the yogurt. Thus resulting in a weakening gel structure with viscosity reduction (Guénard‐Lampron et al. 2020). Similar viscosity decrement during the storage time was observed in lotus leaves fortified yogurts (Kim et al. 2019).

#### 3.3.4 Color of the Fortified Yogurts

**FIGURE 5:**
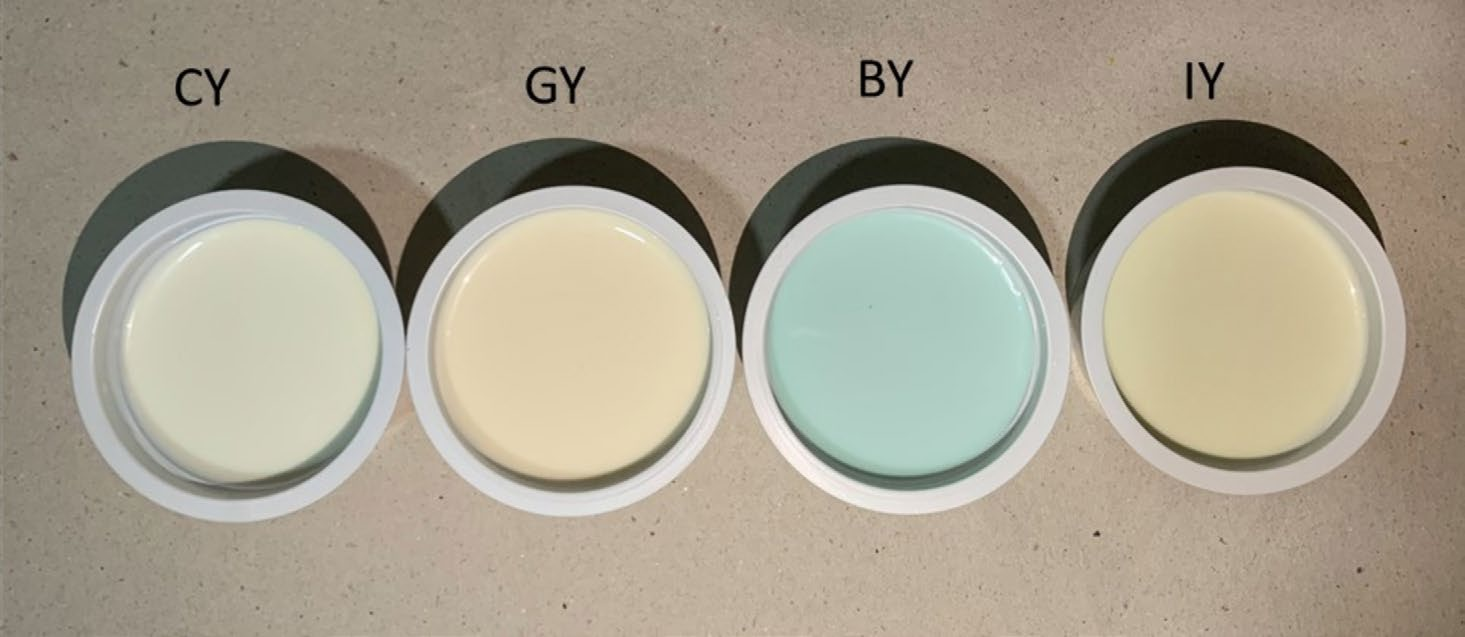
Color of the CY (unfortified yogurt), GY (ginger fortified yogurt), BY (blue butterfly pea flower fortified yogurt), and IY (ivy leaf fortified yogurt) at Day 1.

The visible color of all the yogurts and the changes in the color characteristics of yogurts throughout the storage period are shown in Figure 5 and Table 2, respectively. According to the results, the lightness (L*) of the IY was significantly lowered compared to the CY, while the IY showed a* value similar to the CY on Day 1. In contrast, adding ivy leave extract led to b* of the yogurts significantly on Day 1. This could be attributed to the dark yellow color of the plant extracts due to fiber and hydrocolloid components (Rinaldoni et al. 2012). Moreover, BY exhibited a significantly lower lightness value, which was 10.36% lower than the CY on Day 1. Moreover, it also showed significantly lower a* and b* values compared to CY on Day 1. This could be due to the dark blue anthocyanins of the blue butterfly pea flower (Tashtoush et al. 2016). Furthermore, these color changes were acceptable according to the results of the sensory analysis of this study. Regarding the GY, it did not significantly lower the L*, a*, and b* values compared to the CY on Day 1.

**TABLE 2:** Color parameters analysis of the fortified set yogurts during the 21‐day storage.

Color parameters	Yogurt type	Storage days			
		Day 1	Day 7	Day 14	Day 21
*L*	CY	93.24 ± 1.16^aA^	92.58 ± 0.14^aA^	92.64 ± 0.96^aA^	92.34 ± 0.27^aA^
	GY	92.23 ± 0.23^aAB^	92.36 ± 0.21^aB^	92.17 ± 0.16^aA^	92.30 ± 0.17^aA^
	BY	83.58 ± 0.94^bcC^	84.29 ± 0.17^abC^	84.90 ± 0.12^aC^	83.37 ± 0.66^cC^
	IY	91.41 ± 0.86^aB^	91.54 ± 0.05^aB^	91.39 ± 0.11^aB^	90.71 ± 0.31^bB^
*a*	CY	−1.58 ± 0.4^bA^	−1.26 ± 0.05^aB^	−1.26 ± 0.19^aB^	−1.38 ± 0.07^abC^
	GY	−1.48 ± 0.1^cA^	−1.31 ± 0.11^bB^	−1.11 ± 0.08^aA^	−1.18 ± 0.04^aB^
	BY	−2.21 ± 0.17^cB^	−2.06 ± 0.05^bC^	−1.91 ± 0.03^aC^	−2.04 ± 0.04^bD^
	IY	−1.40 ± 0.09^dA^	−1.12 ± 0.08^cA^	−0.99 ± 0.06^bA^	−0.90 ± 0.00^aA^
*b*	CY	7.19 ± 0.29^aB^	7.13 ± 0.18^aA^	7.34 ± 0.23^dC^	7.19 ± 0.18^aB^
	GY	7.16 ± 0.11^bB^	6.89 ± 0.15^cB^	7.38 ± 0.19^aB^	7.40 ± 0.11^aA^
	BY	−2.83 ± 0.17^dC^	−2.54 ± 0.09^c,C^	−2.03 ± 0.11^aB^	−2.26 ± 0.14^bC^
	IY	7.97 ± 0.19^aA^	6.89 ± 0.11^bB^	7.82 ± 0.10^aA^	7.03 ± 0.07^bB^

*Note:* Each data point represents the mean ± SD (*n* = 9); ^a–d^different letters indicate a significant (*p* < 0.05) difference in the same row, and ^A–D^different letters indicate a significant difference in the same column (*p* < 0.05) (one‐way analysis of variance followed by Tukey post hoc test).

Abbreviations: BY, blue butterfly pea flower fortified yogurt; CY, unfortified yogurt; GY, ginger fortified yogurt; IY, ivy leaf fortified yogurt.

In addition, the lightness of CY, GY, and BY did not change significantly during storage. However, the lightness of the IY was significantly reduced compared with the initial lightness value on Day 1, and it could be associated with the degradation and oxidation of phenolic compounds to develop pheophorbide and pheophytin‐like compounds, which contribute to the dark color formation (Medic et al. 2022). Similar lightness reductions were reported in green tea, moringa leaves, dill fortified yogurts on Day 1 (Shokery et al. 2017; Tizghadam et al. 2021). Moreover, it was observed that the a* value of all the yogurts was significantly increased (increased the redness) over storage, and a* values of all the yogurt samples except BY were significantly higher than that of the CY at the end of the storage. During the storage, the b* value of the CY remained the same as Day 1, while the b* values of GY and BY were significantly increased over the storage. However, the b* value of the IY was significantly lowered over storage. Similarly, date palm, lemon grass, scent leaf, pawpaw leaf, and sorghum stalk‐like extract fortification also exhibited the potential to change the color parameters of yogurts (Almusallam et al. 2021). Moreover, these color changes observed on Day 1 were accepted by the sensory panel according to the sensory results (Figure 1).

### 3.4 Fourier Transform Infrared Spectroscopy (FTIR) Analysis of Fortified Yogurts

**FIGURE 6:**
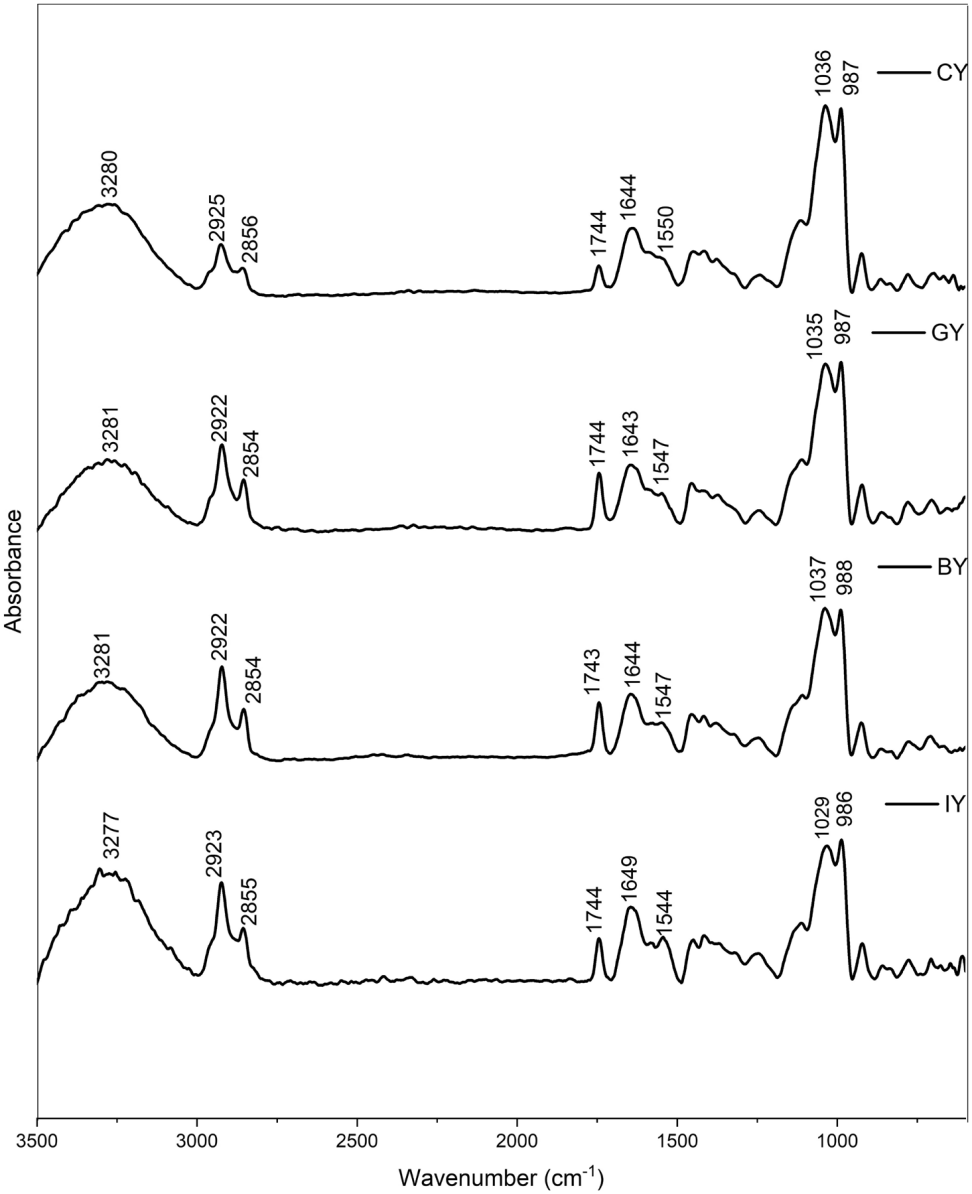
FTIR spectrums of the CY (unfortified yogurt), GY (ginger fortified yogurt), BY (blue butterfly pea flower fortified yogurt), and IY (ivy leaf fortified yogurt).

The obtained FTIR spectra of all the yogurts are shown in Figure 6. According to the results, the yogurts exhibited broad peaks in the region 3250–3300 cm^−1^ related to the stretching vibration of –OH groups in lactose and water molecules (Saberi et al. 2023). IR peaks observed in 2922–2925 cm^−1^ were associated with the symmetric ‐CH_2_ stretching of fatty acids (Saberi et al. 2023). The FTIR peak intensity for fatty acids follows the trend; CY < GY < BY = IY, which can be attributed to differences in the fatty acid content or composition of plant extracts. For instance, ginger contains different fatty acids, including linoleic acid, palmitic acid, and Oleic acid (Oforma et al. 2019). Furthermore, blue butterfly pea flowers consist of phytanic acid and linoleic acid‐like fatty acids (Shen et al. 2016). Although there were no significant differences in fat, as all have approximately 3.1% fat (Table 1), but the different fatty acid compositions (types and quantity) and the sensitivity of the FTIR can be the cause. Similarly, higher peak intensities can be observed at 2855–2856 cm^−1^ compared with the CY, corresponding to methylene in lipids and the asymmetric ‐CH_2_ stretching of methylene chains. This further supports that the introduction of plant materials alters the fatty acid composition in the yogurt (Saberi et al. 2023; Turek et al. 2023).

Furthermore, the peak intensities in IY, BY, and GY yogurts compared with the CY, follow the trend; CY < IY < BY < GY at 1743–1744 cm^−1^. This peak corresponds to the C=O stretching of ester carbonyl groups, which is indicative of triglycerides (Turek et al. 2023). The increasing intensity can be attributed to the introduction of bioactive compounds from the plant materials, which may alter the lipid composition in the fortified yogurts. Moreover, this enhanced peak intensity in fortified yogurts could also be due to the formation of more ester compounds with elevated microbial activity (Liu et al. 2004). In detail, the higher lactic acid bacteria proteolysis casein and produce more amino acids, which are converted to alcohol or carboxylic acids through a series of reactions and form more esters or thio‐esters by catalyzing the reaction between alcohols and carboxylic acid with the esterases or acyltransferases (Mayo et al. 2010). The peaks observed at 1600–1700 cm^−1^ showed the existence of absorbed water and Amide I, which is associated with the milk protein (Jaya 2009). These peaks are related to 80% C = O stretch, 10% C‐N stretch, and 10% N–H bend (Jaya 2009). This milk protein region consists of two major milk proteins such as casein (α‐casein, β‐casein, and κ‐casein) and whey (β‐lactoglobulin, α‐lactalbumin, and serum albumin). Moreover, Lactose typically exhibits high absorptions (C‐O stretching) in the range of 1100–1000 cm^−1^, and IR peaks exhibited at 1029–1037 cm^−1^ confirmed the presence of lactose in all the yogurt samples (Balan et al. 2020). The 986–987 cm^−1^ peaks represented the C‐H bending (Papadopoulou et al. 2021).

**FIGURE 7:**
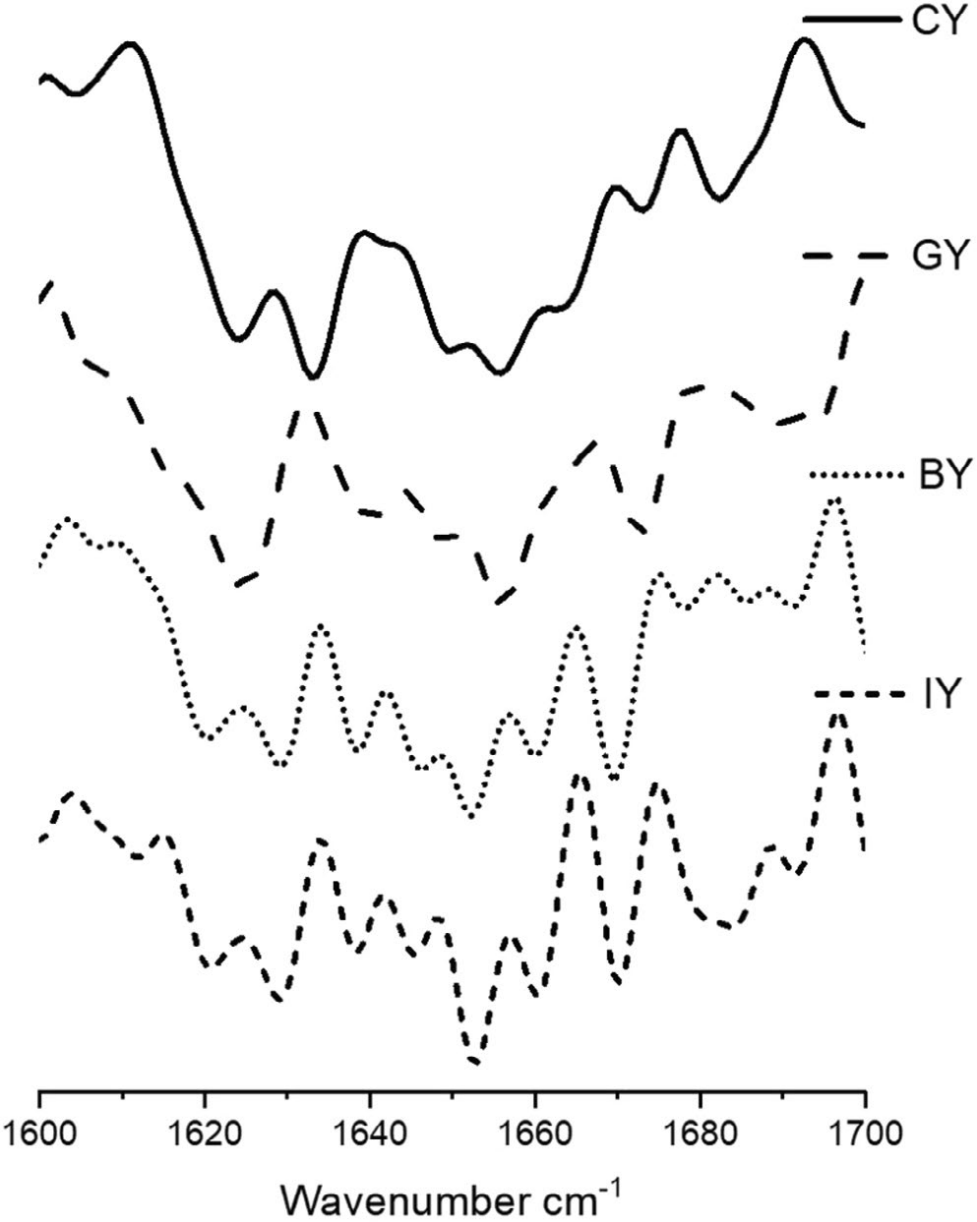
Second derivative of the Amide I region of the CY (unfortified yogurt), GY (ginger fortified yogurt), BY (blue butterfly pea flower fortified yogurt), and IY (ivy leaf fortified yogurt).

According to the secondary structure analysis (Figure 7) of the milk protein, peaks between 1651 and 1653 cm^−1^ represented the α‐helix structure, while large loops are represented in 1656–1658 cm^−1^ in all the yogurts. Furthermore, the peaks between 1619 and 1642 cm^−1^ are assigned to β‐sheets, whereas parallel β‐sheets are assigned to the peaks between 1626 and 1640 cm^−1^ in yogurts. Additionally, the presence of random coil structure has exhibited peaks between 1644 –1648 cm^−1^ (Pelton and McLean 2000; Bogahawaththa et al. 2018). The peaks at 1665^1^–1684 cm^−1^ represented the β‐turns, while 1688–1697 cm^−1^ exhibited the aggregated β‐sheets.

The peak intensities of fortified yogurts increased in β‐sheets at 1624–1642 cm^−1^ could be associated with protein structure refolding and stabilize with plant extracts with phenolic and protein interactions (Wijegunawardhana et al. 2024a). Furthermore, the formation of protein aggregates with the casein precipitation is confirmed by increased random coils at 1644–1648 cm^−1^ (Wijegunawardhana et al. 2024b), and it can be observed that the incorporation of plant extract enhances the protein aggregation in all the fortified yogurts. Furthermore, increased α‐helix peak intensities in GY exhibited strong phenolic and protein interaction. The increase in intensity for loops at 1656 cm^−1^ could be due to the dissociation of κ‐casein from micelles and the establishment of hydrogen bonds between α_S_‐casein and β‐casein with or within the micelle (Markoska et al. 2019). Additionally, the increased β‐turns peak intensities in fortified yogurts suggested the casein participation in forming aggregates. This finding confirms that the fortification of ginger, blue butterfly pea flower, and ivy leaves has a significant impact on protein secondary structure with phenol and protein interactions (Wijegunawardhana et al. 2024a). Similar protein secondary structure changes were observed in the study of phenolic compounds and major whey protein behavior evaluation (Zhang et al. 2014).

Moreover, Lactose exhibits high absorptions (C‐O stretching) in the range of 1100–1000 cm^−1^, and IR peaks exhibited at 1029–1037 cm^−1^ confirmed the presence of lactose in all the yogurt samples (Balan et al. 2020). Furthermore, the highest peak intensity associated with lactose was observed in CY, followed by GY, BY, and IY, respectively. According to findings, microbial growth is inversely proportional to the lactose content (Rodionova et al. 2022) and it can be suggested that the fermentation and the lactose to lactic acid conversion is more efficient with the presence of plant materials. This can be further justified by the pH, acidity (Figure 2A,B), and viable microbial count (Table 3) of the yogurt, which exhibited lower pH levels and higher acidity and microbial count in fortified yogurts than the CY.

**TABLE 3:** Logarithmic count (Log CFU mL^−1^) of the number of *Streptococcus thermophilus* , *Lactobacillus delbrueckii* subsp. *bulgaricus,* and yeast and mold count in fortified yogurts during the 21‐day storage.

Lactic acid bacteria	Yogurt	Storage days			
		Day 1	Day 7	Day 14	Day 21
*S. thermophilus*	CY	8.81 ± 0.01^cC^	8.87 ± 0.00^bB^	8.93 ± 0.01^aB^	8.68 ± 0.02^dA^
	GY	8.82 ± 0.01^bC^	8.82 ± 0.01^bC^	9.03 ± 0.01^aA^	8.56 ± 0.04^cB^
	BY	8.92 ± 0.01^aB^	8.83 ± 0.02^bC^	8.75 ± 0.01^cC^	8.50 ± 0.04^dC^
	IY	8.95 ± 0.01^bA^	8.98 ± 0.01^aA^	8.95 ± 0.01^bB^	8.61 ± 0.03^cB^
*L. delbrueckii* subsp. *bulgaricus*	CY	6.88 ± 0.01^cB^	7.38 ± 0.02^bB^	7.53 ± 0.04^aC^	6.38 ± 0.05^dB^
	GY	6.88 ± 0.01^cB^	7.53 ± 0.02^bA^	7.75 ± 0.01^aA^	6.45 ± 0.03^dA^
	BY	6.75 ± 0.02^aC^	6.76 ± 0.01^aD^	6.66 ± 0.02^bD^	6.32 ± 0.03^cC^
	IY	6.95 ± 0.01^cA^	7.26 ± 0.03^bC^	7.61 ± 0.01^aB^	6.39 ± 0.02^dB^
Yeast and mold	CY	ND	ND	ND	2.19 ± 0.19
	GY	ND	ND	ND	ND
	BY	ND	ND	ND	ND
	IY	ND	ND	ND	ND

*Note:* Each data point represents the mean ± SD (*n* = 9); ^a–d^different letters indicate a significant difference in the same row (*p* < 0.05), and ^A–D^different letters indicate a significant difference in the same column (*p* < 0.05) (one‐way analysis of variance followed by Tukey post hoc test).

Abbreviations: BY, blue butterfly pea flower fortified yogurt; CY, unfortified yogurt; GY, ginger fortified yogurt; IY, ivy leaf fortified yogurt.

### 3.5 Microstructure of the Fortified Yogurts

**FIGURE 8:**
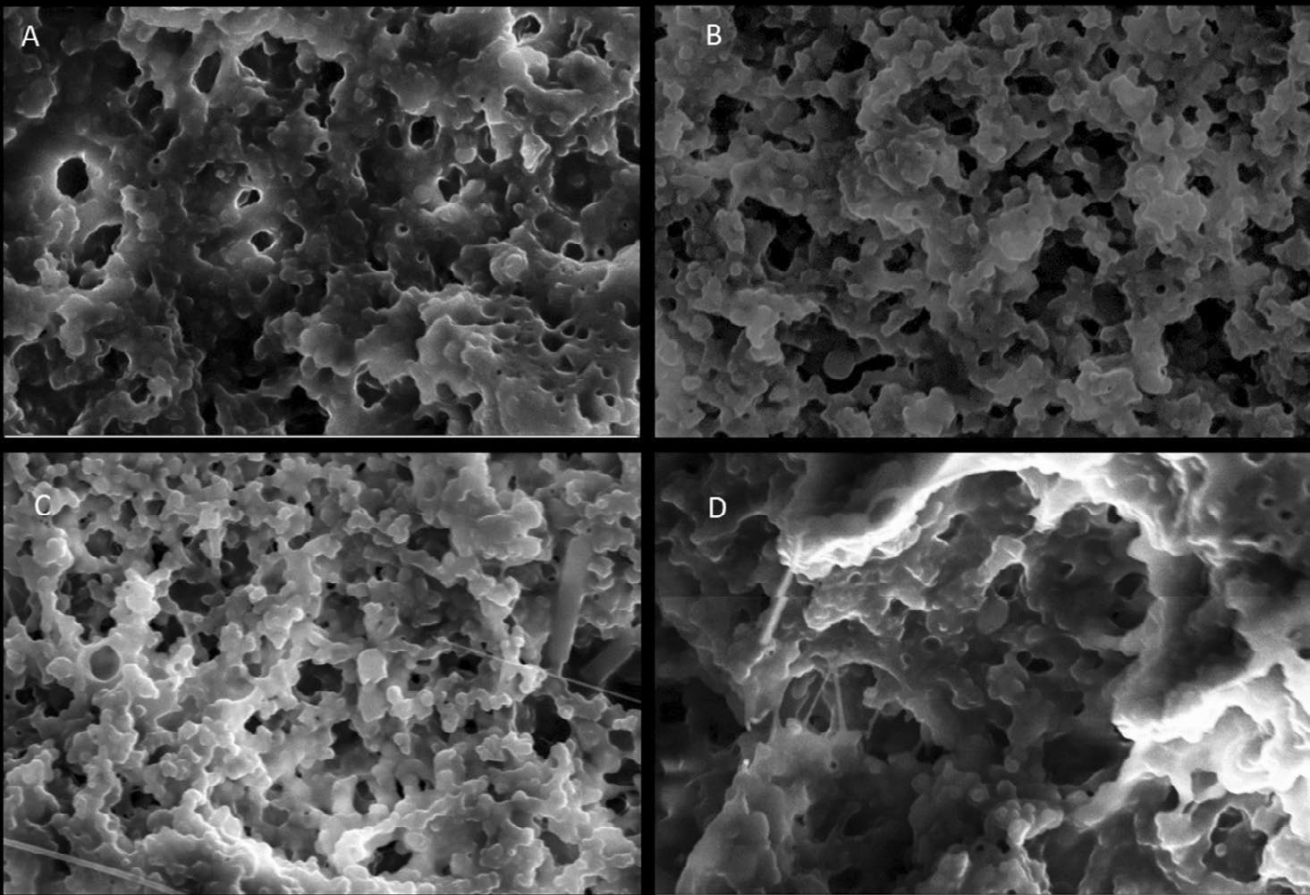
15,000× magnified SEM images of the microstructure of CY (A) (unfortified yogurt), GY (B) (ginger fortified yogurt), BY (C) (blue butterfly pea flower fortified yogurt), and IY (D) (ivy leaf fortified yogurt).

The microstructure of all the fortified yogurts obtained with a scanning electron microscope at a 15,000‐fold magnification is presented in Figure 8. These yogurts exhibited the branched protein structure with many void areas occupied by whey and lactic acid bacteria. Furthermore, it was shown that the fortified plant extract had a substantial impact on the microstructure when comparing the obtained SEM images of the fortified yogurts with CY. The microstructures of the CY (Figure 8A) and IY (Figure 8D) were very coarse, low compact, and low interconnected chains having large granules. Furthermore, IY exhibited a less dense yogurt structure, which consisted of large aggregates compared with the CY. This type of structure caused fast deformation, poor texture parameters, and high syneresis (Domagała 2009). This fact is further supported by the syneresis (Figure 3) and viscosity data (Figure 4) of this study, which exhibited higher syneresis and lower viscosity for IY and CY. However, the addition of ginger Figure 8B and blue butterfly pea flower Figure 8C extract significantly led to a reduction in the protein aggregates size and became more compact and denser. Moreover, GY and BY observed more interconnected chains and small granules of casein micelles joined by thinner fibers. Furthermore, this microstructure reduces the potential of protein rearrangement during storage, which eventually reduces the possibility of syneresis in yogurts (Qiu et al. 2021; Fiszman et al. 1999). Furthermore, this was matched with syneresis results of GY and BY, which showed low syneresis rate and higher viscosity. Similarly, Qiu et al. (2021) reported that fortification of rose extract exhibited a more compact gel structure and exhibited high water‐holding capacity in the yogurts.

### 3.6 Viability of Fermentative Bacteria

The obtained viability counts of fermentative bacteria are shown in Table 3. According to the results of *S. thermophilus* , the aqueous extracts of blue butterfly pea flower and ivy leaf fortification caused a significant increment in the viability of *S. thermophilus* on Day 1 compared with the CY. However, GY exhibited no significant difference in viable count compared to the CY on Day 1. During storage, the *S. thermophilus* count of CY and GY was significantly increased up to Day 14 and then significantly reduced on Day 21, while IY exhibited the viability count increment up to Day 7 and significantly dropped during the rest of the storage period. However, a viability count reduction trend was observed during storage just after Day 1 in BY.

Regarding the viability of *L. delbrueckii* subsp. *bulgaricus*, IY exhibited a significantly higher viability of *L. delbrueckii* subsp. *bulgaricus*, while the BY exhibited a significantly lower viable count on Day 1 compared with the CY. However, GY exhibited no significant difference compared with the CY. As time continued, the viable count of CY, GY, and IY was increased significantly up to Day 14 and significantly reduced thereafter. However, the viable count of BY was significantly reduced after Day 7 and exhibited the minimum viability count at the end of the storage.

The reason for obtaining a higher viability count with the fortification could be due to the available nutrients in the fortifying ingredients, such as fibers and polyphenols, which enhance the metabolic activity of the microorganisms, eventually increasing the growth rate (Dhawi et al. 2020; Abdullah et al. 2023). However, as time went on, the low pH and high acidity conditions could have prevented and slowed down the growth of these bacteria (Jaster et al. 2018; Almusallam et al. 2021). A similar viability count reduction during storage was observed in moringa and fenugreek‐fortified yogurts, oats, and raspberry‐fortified yogurts (Dhawi et al. 2020; Savas and Akan 2021). However, the observed *S. thermophilus* viable count reduction over storage in BY after Day 1 could be due to the formation of toxic compounds by the degradation of phenolic compounds in blue butterfly pea flower extracts during fermentation (Kynadi and Suchithra 2017).

However, the sum of both lactic acid bacteria's viable counts of all the yogurts was above 7 Log CFU mL^−1^ throughout the storage period, which was acceptable with the Alimentarius Codex specification (Codex 2011) that states 7 Log CFU mL^−1^ as the minimum viable count for yogurts.

The yeast and mold count of the yogurt is a significant indicator that can be used to evaluate its hygiene quality and shelf life. However, yeast and mold were not detected throughout the storage period in all the fortified yogurts and exhibited acceptable shelf life until Day 21 (Table 3). This could be associated with the antimicrobial properties of different phytochemicals found in plant extracts, including alkaloids, flavonoids, tannins, and saponins (Kha and Le 2021). Moreover, the yeast and mold count was not detected in the CY until Day 14. However, on Day 21, 2.19 ± 0.19 Log CFU mL^−1^ was observed in the CY, though it was an acceptable level according to the SLSI guidelines, which indicate that the yeast and mold count should be < 10 CFU mL^−1^. Similarly, a study found that coriander leaf powder‐fortified yogurt was free from yeast and molds, while unfortified yogurt showed yeast and mold growth over storage (Gundogdu et al. 2020).

### 3.7 Total Phenolic Content and Antioxidant Properties of Fortified Yogurt

**FIGURE 9:**
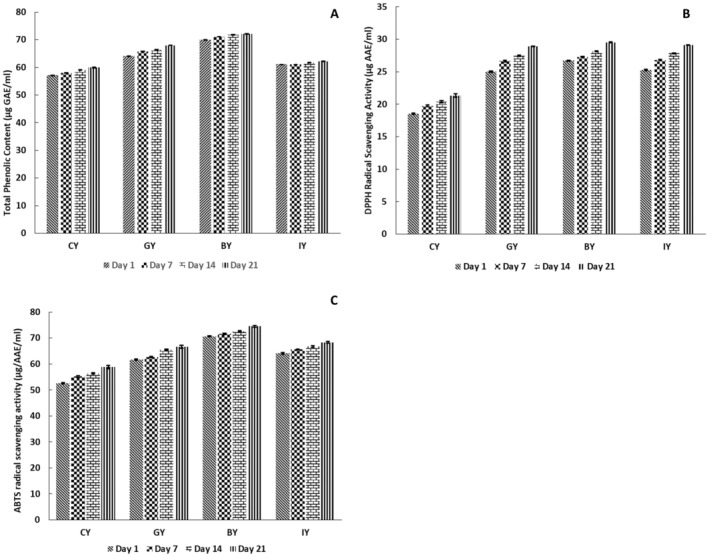
Total phenolic content (A) (μg Gallic acid equivalent mL^−1^), DPPH radical scavenging activity (B) (μg Ascorbic acid equivalent mL^−1^), and ABTS radical scavenging activity (C) (μg Ascorbic acid equivalent mL^−1^) in CY (unfortified yogurt), GY (ginger fortified yogurt), BY (blue butterfly pea flower fortified yogurt), and IY (ivy leaf fortified yogurt) during the 21‐day storage (one‐way analysis of variance followed by Tukey post hoc test).

The results of the total phenolic content, DPPH, and ABTS radical scavenging activities of the fortified yogurts are shown in Figure 9A–C, respectively. The total phenolic content and antioxidant activity of GY, BY, and IY significantly improved on Day 1 compared with the CY. The increased total phenolic and antioxidant activity with the plant extract fortification could be associated with the availability of specific phytochemicals such as phenolic and flavonoid compounds (Akan et al. 2022). Especially, ginger contains gingerols, shogaols, paradols, and gingerdions‐like compounds; blue butterfly pea flower contains delphinidin derivatives, rutin, and ternatins‐like compounds; and ivy leaves contain gallic acid, kaempferol, quercetin, rutin, ferulic acid, and caffeic acid‐like compounds (Nair et al. 2015; Al‐Madhagy et al. 2019; Ghafoor et al. 2020). These compounds could contribute to the total phenolic and antioxidant content of fortified yogurts. Similarly, Aamir et al. (2023) reported that fortification of ginger powder significantly increased the total phenolic content of the yogurts compared to unfortified yogurts, though the content was lower than that observed in the present study. In contrast, fortification of 1% ginger powder to yogurts showed higher total phenolic content compared with this study (Felfoul et al. 2017), and this could be due to the processing conditions, analytical methods, extraction method, environmental conditions, and calibration procedures (Michiu et al. 2022). Regarding the storage study, all the yogurts exhibited total phenolic content and antioxidant activity, which improved the trend over storage. This could be attributed to the peptides produced by the breakdown, which are generated by microbial activity. These peptides and free amino acids react with DPPH and ABTS‐like reagents, reducing their reactivity and indicating increased antioxidant activity in the yogurt (Chalid et al. 2021). Moreover, p‐hydroxybenzoic acid and vanillic acid‐like phenolic acids produced during the action of lactic acid bacteria fermentation in yogurt also support the enhancement of available total phenolic and antioxidant activity during storage (Blum 1998). This antioxidant enhancement could be more beneficial for quick wound healing in the human body, has anti‐aging properties, and prevents the risk of cardiovascular diseases and cancers (Martemucci et al. 2022).

## 4 Conclusion

In this study, 2% ginger, 2% ivy leaves, and 3% blue butterfly pea flower aqueous extracts fortified yogurts showed better properties. Results revealed that these plant extracts could potentially improve sensory qualities even though they were insignificant. Additionally, ginger, ivy leaf, and blue butterfly pea flower aqueous extracts improved the viability of lactic acid bacteria significantly. It promoted a lower pH value and higher acidity conditions in fortified yogurts without altering the basic nutrition profile. Furthermore, fortification of ginger and blue butterfly pea flower extracts reduced the protein aggregate size and formed a denser gel structure, thus improving the viscosity and water‐holding capacity. In contrast, adding ivy leaf extract decreased water‐holding capacity and viscosity in the fortified yogurts. Additionally, GY, BY, and IY can enhance the antioxidant and total phenolic content, and incorporating these plant extracts into yogurt could offer more significant health benefits. Thus, ginger and blue butterfly pea flowers can be introduced to create value‐added yogurt products with enhanced sensory appeal and functional benefits. These findings could be beneficial for the future commercialization of plant extract‐fortified functional yogurt as a product that attracts consumers' preference due to properties such as the ability to achieve a superior yogurt texture, minimize the use of additional additives for this process, and maintain stability throughout its shelf life.

## Author Contributions

**Yohan Jayaweera:** formal analysis (equal), investigation (equal), methodology (equal), writing – original draft (equal). **Mayumi Silva:** conceptualization (equal), funding acquisition (equal), project administration (equal), supervision (equal), validation (equal), writing – review and editing (equal). **Jayani Chandrapala:** supervision (equal), validation (equal), writing – review and editing (equal). **Rumesh Liyanage:** supervision (equal), validation (equal), writing – review and editing (equal). **K. G. L. R. Jayathunge:** supervision (equal), validation (equal), writing – review and editing (equal). **Amali U. Alahakoon:** conceptualization (equal), funding acquisition (equal), project administration (equal), supervision (equal), writing – review and editing (equal).

## Ethics Statement

The authors have nothing to report.

## Consent

Written informed consent was obtained from all study participants.

## Conflicts of Interest

The authors declare no conflicts of interest.

## Data Availability

Data will be made available on request.
